# Single-step ethanol production from raw cassava starch using a combination of raw starch hydrolysis and fermentation, scale-up from 5-L laboratory and 200-L pilot plant to 3000-L industrial fermenters

**DOI:** 10.1186/s13068-021-01903-3

**Published:** 2021-03-16

**Authors:** Morakot Krajang, Kwanruthai Malairuang, Jatuporn Sukna, Krongchan Rattanapradit, Saethawat Chamsart

**Affiliations:** 1 Biological Science Program, Faculty of Science, Burapha University, Chon Buri, 20131 Thailand; 2 Department of Biology, Faculty of Science, Burapha University, Chon Buri, 20131 Thailand; 3 Biochemical Engineering Pilot Plant, Faculty of Science, Burapha University, Chon Buri, 20131 Thailand; 4 Department of Biotechnology, Faculty of Science, Burapha University, Chon Buri, 20131 Thailand

**Keywords:** Bioethanol, Single-step ethanol production, Raw cassava starch, Hydrolysis, Fermentation, Pilot scale, Industrial scale

## Abstract

**Background:**

A single-step ethanol production is the combination of raw cassava starch hydrolysis and fermentation. For the development of raw starch consolidated bioprocessing technologies, this research was to investigate the optimum conditions and technical procedures for the production of ethanol from raw cassava starch in a single step. It successfully resulted in high yields and productivities of all the experiments from the laboratory, the pilot, through the industrial scales. Yields of ethanol concentration are comparable with those in the commercial industries that use molasses and hydrolyzed starch as the raw materials.

**Results:**

Before single-step ethanol production, studies of raw cassava starch hydrolysis by a granular starch hydrolyzing enzyme, StargenTM002, were carefully conducted. It successfully converted 80.19% (w/v) of raw cassava starch to glucose at a concentration of 176.41 g/L with a productivity at 2.45 g/L/h when it was pretreated at 60 °C for 1 h with 0.10% (v/w dry starch basis) of Distillase ASP before hydrolysis. The single-step ethanol production at 34 °C in a 5-L fermenter showed that *Saccharomyces cerevisiae* (Fali, active dry yeast) produced the maximum ethanol concentration, *p*_max_ at 81.86 g/L (10.37% v/v) with a yield coefficient, *Y*_*p/s*_ of 0.43 g/g, a productivity or production rate, *r*_*p*_ at 1.14 g/L/h and an efficiency, Ef of 75.29%. Scale-up experiments of the single-step ethanol production using this method, from the 5-L fermenter to the 200-L fermenter and further to the 3000-L industrial fermenter were successfully achieved with essentially good results. The values of *p*_max,_ *Y*_*p/s*_, *r*_*p*_, and Ef of the 200-L scale were at 80.85 g/L (10.25% v/v), 0.42 g/g, 1.12 g/L/h and 74.40%, respectively, and those of the 3000-L scale were at 70.74 g/L (8.97% v/v), 0.38 g/g, 0.98 g/L/h and 67.56%, respectively. Because of using raw starch, major by-products, i.e., glycerol, lactic acid, and acetic acid of all three scales were very low, in ranges of 0.940–1.140, 0.046–0.052, 0.000–0.059 (% w/v), respectively, where are less than those values in the industries.

**Conclusion:**

The single-step ethanol production using the combination of raw cassava starch hydrolysis and fermentation of three fermentation scales in this study is practicable and feasible for the scale-up of industrial production of ethanol from raw starch.

## Background

The “hydrolysis and fermentation” of starch to bioethanol is widely employed for the production of biofuel, pharmaceutical and cosmetic ethanol, potable alcohols, e.g., beer, whiskey, other distilled spirits, and other ethanol products. The production of fuel ethanol from starch was first introduced in the United States at the beginning of the twentieth century [1]. Bioethanol is an alternative energy source to replace the utilization of those fuels. Bioethanol is an attractive alternative fuel because it is an eco-friendly renewable bio-based resource contributing to the reduction of fuel emissions that affect the climate change and the negative environmental impacts generated by the worldwide utilization of petroleum oil [2–4]. Bioethanol is the major source of renewable biofuels with about 110 billion liters (BL) produced in 2019 [1, 5], mainly obtained from corn starch and sugarcane. The bulk of the bioethanol of 59.8 BL in 2019 produced in the USA is primarily from corn starch, using about 205 operational plants [5].

Starch is the first generation (1G) feedstock that is the most abundant renewable carbon source and is more readily digestible for conversion to biofuels than cellulosic second generation (2G) feedstock. However, starchy corn gains the lowest ethanol yield per unit area of cultivation when compared to that of other crops. Bioethanol can be produced from various kinds of feedstock. However, when ethanol yields per unit area of cultivations are compared, cassava is the highest potential crop to gain the highest yield. Average yields of cassava, sugar beet, sweet potato, wheat, sugar cane, rice, sorghum, and starchy corn are about 31.25, 56.00, 30.00, 9.00, 62.50, 7.31, 6.25, and 6.00 MT/ha/year (metric ton/ha/year), respectively. Carbohydrate contents (as starch or sugar) of those crops are approximately 28.0, 14.0, 24.5, 70.0, 10.5, 80.0, 70.0, and 70.0 (% w/w), respectively. Theoretical yields of bioethanol per unit area of cultivation per annum of those crops could be 4.95, 4.44, 4.16, 3.57, 3.53, 3.31, 2.48, and 2.38 MT/ha/year (tons of ethanol/ha/year), respectively. They were calculated from a ton of starch produces 566 kg of ethanol, and a ton of sucrose sugar produces 538 kg of ethanol (see Additional file 1).

Cassava is the major energy crop and one of the renewable resources that is utilized for bioethanol production. Cassava starch can be used at large scales to produce ethanol in tropical countries where Thailand is one of the largest cassava producers in the world [6]. The global market stood at 6.90 million MT in 2019. It is a renewable carbohydrate carbon source and available in very abundance. It is cheap (450 US $/MT), clean, non-toxic, and widely used as the important feedstock for various industrial applications especially for the production of ethanol [7]. Therefore, bioethanol production from starch has been extensively researching [8] and still need more developments for more effective industrial productions with higher efficiencies.

Our research work is herein pertinent with the “Recent Advances” addresses [1] in June 2020 for starch-to-ethanol conversion providing a platform for the development of raw starch consolidated bioprocessing (CBP) technologies. Several proof-of-concept studies identified the improved enzyme combinations, alternative feedstocks, and novel strains [9, 10] for evaluation and application under fermentation conditions. In their reviews, different CBP approaches were defined, discussed, and also highlighted the role of enzymes for supplemented CBP processes. Various achievements of amylolytic *Saccharomyces cerevisiae* strains [9, 10] for CBP of raw starch and the remaining challenges that need to be tackled/ pursued to bring yeast raw starch CBP to industrial realization were described [1]. Most of the advances on raw starch CBP have resulted from small batches (50 to 100-mL volumes) or just bioreactor-scale (1 to 2-L volumes) studies and therefore only represent a proof-of-concept. However, further research efforts are required before this technology can be scaled-up to an industrial level. The scale-up of raw starch CBP processes at the industrial level remains an important hurdle to progress to commercialization [1].

At present, the conventional large-scale ethanol production from starch is a batch process [7] comprises three steps: (i) liquefaction by alpha-amylase to reduce the viscosity of the starch and to fragment in the starch chains to the small-sized fragments, followed by (ii) saccharification whereby the liquefied starch is hydrolyzed in to fermentable sugar, i.e., glucose using glucoamylase. Finally, (iii) the glucose is fermented to ethanol by yeast cells [11, 12]. In the process to convert starch to ethanol, starch granules must be gelatinized and liquefied at a high temperature before saccharification and fermentation [13]. The conventional enzymatic liquefaction and saccharification of starch have many disadvantages. They require high-energy inputs [14] including enormous amounts of steam and efficient water-based cooling systems to bring down the temperatures for fermentation [15], thus increasing the production costs of starch-based ethanol. Besides the conventional process of ethanol production, the simultaneous saccharification and fermentation (SSF) process has been widely used [16]. After liquefaction, saccharification is performed simultaneously with the fermentation. This process uses glucoamylase and free cells of yeast at the same time in a single fermenter. The advantages of the SSF process include the reduction of cost because less equipment and fermentation time are required, resulting in higher ethanol productivity and profitability [17, 18]. However, SSF processes also have significant impacts on energy consumption because liquefaction steps are operated at high temperatures.

The conventional starch liquefaction and saccharification processes are energy-intensive, complicated, and not environmentally friendly. Therefore, the processes to reduce high energy consumption are required. If the hydrolysis of starch at a gelatinization temperature was avoided, the costs of 30–40% due to the high energy consumption of starch-based ethanol in the manufacturing process can be saved [19]. The direct hydrolysis of raw starch to glucose by raw starch-digesting glucoamylase at a low temperature is so-called the “cold process”, which significantly simplifies processing and reduces the cost of producing starch-based products [20], e.g., bioethanol and other bioproducts. The cold process saves on energy costs, as well as 40–50% of the total capital and the operational costs [21].

The literature review of recent research updates is addressed here. For bioethanol production based on the cold process, large companies developed novel and efficient enzymes for the saccharification of starch at lower temperatures. Genencor International Inc. (now DuPont) released STARGEN™ and Novozyme released the BPX™ for cold processes [1]. *S. cerevisiae* IR2 was immobilized in the reservoir and the system was used for simultaneous amylase production, hydrolysis and ethanol production from raw cassava starch. The process was very stable for more than seven batches providing an ethanol concentration of 90 g/L with a yield coefficient of 0.46 g/g and a productivity of 1.73 g/L/h [22]. A single-step ethanol production by co-cultures of amylolytic fungus and *S. cerevisiae* TISTR 5088 was studied. The most effective fungus could convert starch at the concentrations of 20% and 25% to fermenting sugar at 115.94 and 159.72 g/L, respectively. In a co-culture system, ethanol at a concentration of 7.37% (w/v) was obtained from using cassava starch medium at a concentration of 20% (w/v) with 65.11% of theoretical yield (% efficiency) [23]. The amylolytic *S. cerevisiae* strains displayed improved fermentation vigor on raw corn starch and broken rice, reaching 97% efficiency and converting 100% of the available carbon to products within 120 h in small-scale CBP fermentations on broken rice [24]. Ethanol at a concentration of 10.22% (w/v) with 78% efficiency was obtained from modified SSF using co-fermentation of the enzymatic hydrolysate of 300 g raw cassava chips/L with cane molasses [25]. The cold hydrolysis of cassava pulp (CP) and its use in SSF to produce ethanol were undertaken. The cold hydrolysis at 50 °C for 2 h, followed by at 30 °C for 72 h gave satisfactory saccharification result. Its further SSF yielded ethanol at a concentration of 27.4 g/L, a yield coefficient on CP of 0.27 g/g with 57.8% efficiency [26]. A single-step ethanol production from raw cassava starch by *K. marxianus* SS106 in 5-L stirred tank fermenter by cold hydrolysis process was conducted. Ethanol at a concentration of 6.17% w/v with a productivity of 0.86 g/L/h was obtained [27].

To overcome the high-temperature-cooking fermentation in the industrial ethanol production from cassava starch, a single-step ethanol production process of simultaneous raw starch hydrolysis and ethanol fermentation in a single fermenter was undertaken in this study. This process is not only yeast fermentation, but also includes simultaneous hydrolyzation of raw cassava starch at a low temperature by the addition of a mixture of raw starch-digesting enzymes at the initial stage, which significantly decreases the energy consumption and the operation cost. Moreover, the low-temperature fermentations have been conducted at the pilot-plant-scale and industrial-scale productions without contamination by bacterial cells and yielded the fermentation efficiencies similar to those of the conventional fermentations. Another advantage of the single-step process is able to maintain a low concentration of glucose during fermentation, which could decrease the inhibitory effects of glucose on the enzyme and yeast activities [11]. This results in minimizing the major by-products, i.e., glycerol, lactic acid, and acetic acid.

The objective of this study is to combine both raw cassava starch hydrolysis and ethanol fermentation in a single process step by utilizing the cold enzymes which are capable of hydrolyzing raw cassava starch under fermentation conditions. This reduces the complexities of operations by a combination of raw cassava starch hydrolysis and fermentation in a single step and single fermenter. It results in saving on high energy consumption, operation cost, and time. This study aims to optimize the hydrolysis of raw cassava starch by the enzyme StargenTM002 and to develop a practicable single-step ethanol production process using raw cassava starch as the raw material at the laboratory, the pilot, and the industrial scales.

## Results and discussion

### The optimization of enzyme StargenTM002 conditions for raw cassava starch hydrolysis

Formations and degree of conversions of glucose from raw cassava starch hydrolyses by varying concentrations of the enzyme StargenTM002, temperatures, and pH values, for those optimal values are shown in Table 1. Considering the enzyme dosages, it was found that the highest glucose concentration was achieved by using StargenTM002 at a concentration of 0.30% (v/w ds). A 200 g/L of raw cassava starch with the enzyme dosage at 0.30% (v/w ds) yielded the glucose at a concentration of 73.78 g/L with 33.54% (w/w) degree of starch conversion. In order to assess the effect of temperature on raw cassava starch hydrolysis, the hydrolyzations were carried out at 30, 35, and 40 °C. There was an increase in quantities of glucose released and higher degrees of conversion of raw starch to glucose when the temperature of starch slurry was increased from 30 to 40 °C. The hydrolysis at 40 °C gained the highest glucose concentration of 133.27 g/L with 60.58% (w/w) degree of conversion. Further, pH values of raw starch slurries from pH 3.0*–*7.0 were verified for their effects on raw starch hydrolyses. Results showed that glucose concentrations and degrees of conversion were increased when pH values of starch slurry were decreased from 7.0 to 3.0. The starch hydrolysis by StargenTM002 at pH 3.0 yielded the maximum glucose concentration of 114.39 g/L with 52% (w/w) degrees of conversion. However, this experiment was concerned that the pH value below 4.0 could be a negative effect on yeast cell growth and fermentation activities. The pH is one of the most important parameters influencing yeast cell growth and fermentation activities. Low initial pH values cause chemical stress on yeast cells affecting the accumulated biomass loss, the decrease in consumption rate of sugar, the decrease in final concentration of ethanol, and the increase in final concentrations of glycerol [28, 29]. Several studies investigating the influence of pH on *S. cerevisiae* fermentations have been published. A pH at 4.5 gave the highest ethanol production from *S. cerevisiae* [30]. A pH below 3.5 led to reduce yeast viability and its vigor as well as lower ethanol yield [31]. Optimal pH values for yeast growth could vary from 4.0 to 6.0, depending on their strains and the decrease in ethanol production was observed when the initial medium pH was at 3.0 [32]. The pH is considered an important factor for survival and growth of yeasts. It affects the permeability of the cell membrane and on the enzymes that are active in degrading the substrate [33]. Therefore, in our further studies, the pH value at 4.0 was used as the optimum pH on raw starch hydrolysis and ethanol fermentation.

**Table 1:** Release of glucose, productivity, and degree of conversion of raw cassava starch to glucose from raw cassava starch hydrolyses by StargenTM002 at various enzyme dosages, temperatures, and initial pH values

Main factors	Glucose concentration (g/L)	Productivity, *r*_*p*_ (g/L/h)	Degree of conversion to glucose (%w/w)
StargenTM002 (% v/w)			
0.1	37.45 ± 5.98^c^	0.99 ± 0.27^c^	17.02 ± 2.72^c^
0.2	56.56 ± 1.36^b^	1.18 ± 0.03^b^	25.71 ± 0.62^b^
0.3	73.78 ± 4.18^a^	1.54 ± 0.09^a^	33.54 ± 1.90^a^
0.4	78.70 ± 1.41^a^	1.64 ± 0.03^a^	35.77 ± 0.64^a^
Temperature (°C)			
30	30.07 ± 0.39^c^	0.63 ± 0.01^c^	13.67 ± 0.18^c^
35	59.50 ± 5.31^b^	1.24 ± 0.11^b^	27.05 ± 2.41^b^
40	133.27 ± 7.83^a^	2.78 ± 0.16^a^	60.58 ± 3.56^a^
Initial pH			
3.0	114.39 ± 1.24^a^	2.38 ± 0.03^a^	52.00 ± 0.56^a^
4.0	96.39 ± 1.03^b^	2.01 ± 0.02^b^	43.82 ± 0.47^b^
5.0	56.26 ± 1.17^c^	1.17 ± 0.02^c^	25.57 ± 0.53^c^
6.0	33.10 ± 1.14^d^	0.69 ± 0.02^d^	15.04 ± 0.52^d^
7.0	16.59 ± 0.36^e^	0.35 ± 0.01^e^	7.70 ± 0.16^e^

Statistic comparisons of those mean values within their own columns (among main factors) at *p*-values ≤ 0.05 show different characters, a, b, c, d, and e, which indicate statically significant differences

### Raw cassava starch pretreatments and hydrolyses

Formations and degrees of conversion of glucose from raw cassava starch hydrolyses by enzyme StargenTM002 at the optimum conditions for 72 h are shown in Table 2. It was found that the hydrolysis of raw cassava starch at pH 4.0 and 40 °C for 72 h with StargenTM002 at a concentration of 0.3% (v/w ds) generated the glucose at a concentration of 150.83 g/L with 68.56% (w/w) degree of conversion. This result indicated that it was incomplete hydrolysis. Consequently, the pretreatment of raw cassava starch which influences the StargenTM002 activity is an interesting strategy for increasing the hydrolysis capability.

**Table 2:** Release of glucose, productivity, and degree of conversion of starch to glucose from pretreatments of raw cassava starch and further hydrolyses by StargenTM002 at optimum conditions for 72 h

Starch slurry pretreatment (1 h)			Hydrolysis by StargenTM002 (40 °C, 72 h)		
Temperature (°C)	Distillase ASP (% w/w)	Urea (% w/w)	Glucose concentration (g/L)	Productivity, *r*_p_ (g/L/h)	Degree of conversion to glucose (% w/w)
Non-pretreatment treatment			150.83 ± 1.58^d^	2.09 ± 0.02^d^	68.56 ± 0.72^d^
60	–	–	159.90 ± 2.85^c^	2.22 ± 0.04^c^	72.68 ± 1.30^c^
60	0.1	–	176.41 ± 1.52^a^	2.45 ± 0.02^a^	80.19 ± 0.69^a^
60	0.2	–	173.81 ± 2.28^a^	2.41 ± 0.07^a^	79.00 ± 1.20^a^
60	0.3	–	175.93 ± 1.99^a^	2.44 ± 0.02^a^	79.97 ± 1.81^a^
60	0.1	1.0	167.81 ± 1.51^b^	2.33 ± 0.02^b^	76.28 ± 0.68^b^
60	0.1	2.0	171.55 ± 8.09^b^	2.38 ± 0.11^b^	77.93 ± 3.68^b^
60	0.1	3.0	166.47 ± 1.15^b^	2.31 ± 0.02^b^	75.67 ± 0.52^b^

Statistic comparisons of those mean values within their own columns (among slurry pretreatments) at *p*-values ≤ 0.05 show different characters, a, b, c, and d, which indicate statically significant differences

Table 2 shows glucose concentrations, productivities, and degrees of conversion of starch to glucose from pretreatments of raw cassava starch by heat, enzyme Distillase ASP, or urea, and subsequently followed by hydrolyses with StargenTM002 at the optimum conditions. After the raw cassava starch pretreated by heat at a sub-gelatinization temperature of 60 °C for 1 h, the starch slurry was further hydrolyzed by StargenTM002 at the optimum conditions. Results showed that sub-gelatinization temperature pretreatment increased the raw cassava starch hydrolysis activity by StargenTM002. Compared with the non-pretreatment treatment, the heat at 60 °C generated a higher glucose concentration of 159.90 g/L with 72.68% (w/w) degree of conversion.

Moreover, the pretreatment of raw cassava starch at the sub-gelatinization temperature of 60 °C together with Distillase ASP increased glucose formation and its degree of conversion. The highest glucose concentration of 176.41 g/L with a 80.19% (w/w) degree of conversion was successfully achieved when raw cassava starch was pretreated at 60 °C together with 0.10% (v/w ds) of Distillase ASP before the step of StargenTM002 hydrolysis. However, increases in Distillase ASP dosages to 0.20 and 0.30 (% v/w ds) for the pretreatments did not significantly increase further raw starch hydrolyses by StargenTM002.

On the contrary, the heat pretreatment at 60 °C together with Distillase ASP plus urea at the concentrations of 1.0*–*3.0% (w/w sd) did not significantly affect raw cassava starch hydrolyses when compared with the pretreatment without urea. This result indicates that urea pretreatment does not improve raw cassava starch hydrolysis by StargenTM002. This studied result does not agree well with [34] who reported that the combination of urea addition and sub-gelatinization temperature pretreatment greatly improved triticale and corn starch hydrolyses by breaking hydrogen bonds in starch molecules. The reason for the difference between this result and those of [34] are unclear, but one of the reasons is the difference in starch structures of cassava to those of triticale and corn.

**Fig. 1:**
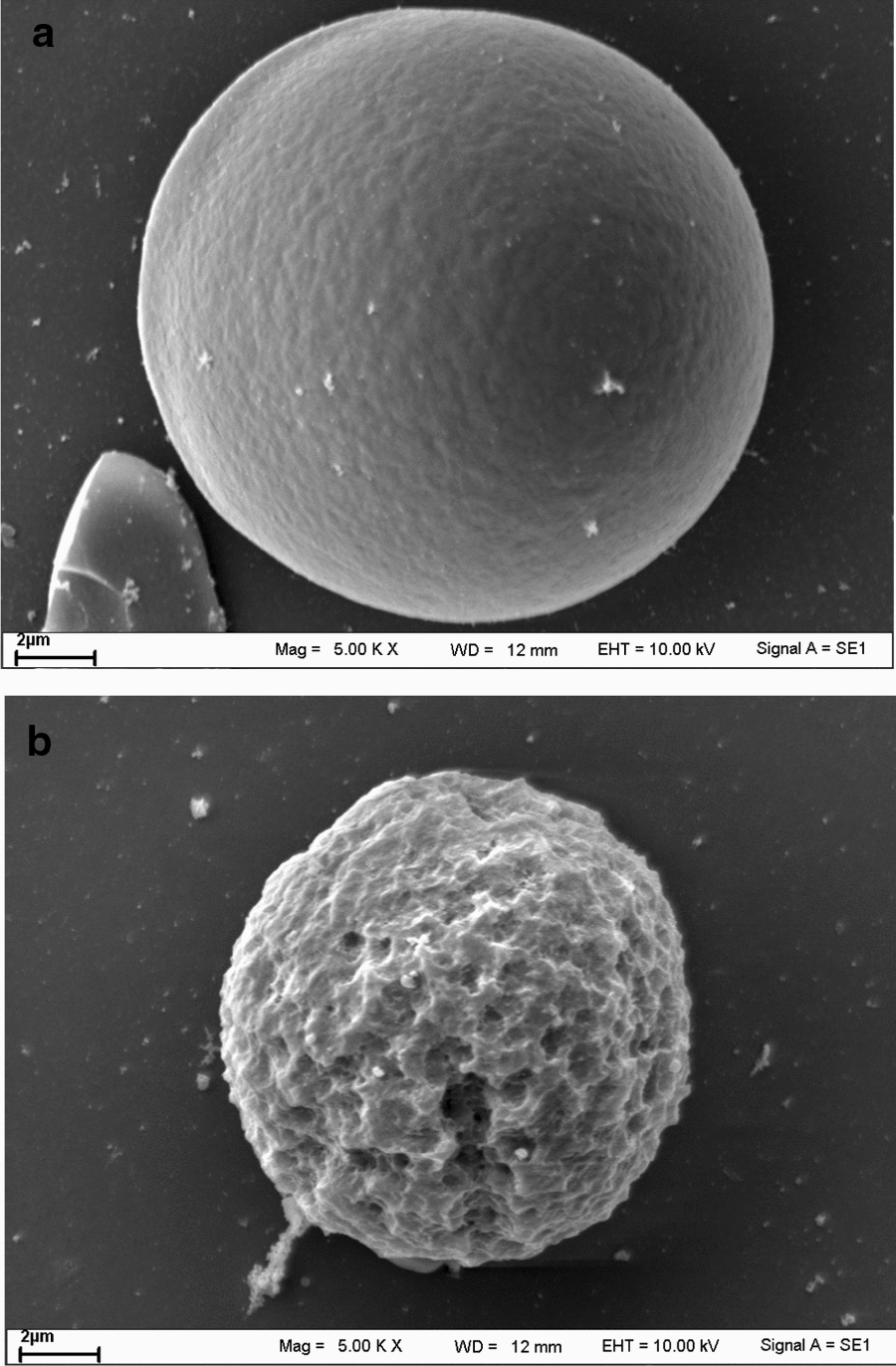
Scanning electron micrographs: **a** pretreated raw cassava starch granule at a sub-gelatinization temperature of 60 °C with Distillase ASP for 1 h and **b** hydrolyzed raw cassava starch granule by StargenTM002 for 48 h

Morphological and microstructural changes of the pretreated and hydrolyzed raw cassava starch granules revealed by a scanning electron microscope (SEM) are shown in Fig. 1. From SEM micrographs, the surface of the pretreated raw cassava starch granules at 60 °C together with 0.10% (v/w ds) enzyme Distillase ASP for 1 h was smooth with few furrows and shallow depressions (Fig. 1a). The hydrolyzation of raw cassava starch granules by StargenTM002 for 48 h resulted in the degradation of most starch to glucose fermentable sugar. Many large enlarged surface holes were observed on the residual starch granule (Fig. 1b).

Sub-gelatinization temperature pretreatment would allow the starch granules to swell and open up the pore on the granule surfaces [14] which increased the ability of Distillase ASP to hydrolyze starch granule surface resulting to increase surface area for later StargenTM002 attack. The raw cassava starch hydrolysis by StargenTM002 was initiated from the granule surface by size enlargement of existing holes [35]. Moreover, the roughened surface in hydrolyzed raw cassava starch granule might be due to the uneven shortening of amylopectin molecules by the action of amylase enzyme [7]. A sub-gelatinization temperature pretreatment causes the starch granule to swell and Distillase ASP action later increases the starch granules surface, resulting in the StargenTM002 to penetrate into the granule more extensively forming pits and channels during hydrolysis. The enzyme degraded the external part of the starch granule by exo-corrosion as holes. These results were also in accordance with [34, 36] who reported that enzymatic corrosion occurred mainly from the cassava starch granule surface to the center. The rough surface and corroded granules were observed in hydrolyzed heat-treated starch compared to hydrolyzed native starch which displayed rough surface with limited erosion and fewer holes [14]. Enzymes are adsorbed on the surface of starch granule and induce holes on the surface where glucose is released [37]*.*

Results from the previous study showed uncompleted hydrolysis of raw cassava starch by StargenTM002 (Table 2). The degree of hydrolysis slightly increased after washing the residual starch and adding a fresh enzyme dosage. No rapid hydrolysis was observed after removing the enzyme by washing and adding a new amylase solution [38]. Therefore, it was assumed that the raw cassava starch hydrolysis was uncompleted due to the presence of a residue of resistant starch. Using the X-ray diffraction patterns could differentiate between the native and the hydrolyzed starch by detection of the change in crystallinity of granular starch. Crystalline types of native and hydrolyzed cassava starch were not markedly changed. However, the crystalline peak of hydrolyzed starch became bigger when compared with that of the native starch. The amorphous region of the granule was hydrolyzed more extensively than the crystalline region [14, 34]. Thus, in this study, the hydrolysis might primarily occur in the amorphous regions of the starch granules. When StargenTM002 hydrolyzed the starch granules, it could primarily degrade the amorphous regions. The crystalline structure might increase the raw cassava starch residue.

### Single-step ethanol production in 5-L laboratory fermenter using combination of raw cassava starch hydrolysis and fermentation

Single-step ethanol fermentations by *S. cerevisiae* (Fali) were conducted in the 5-L fermenters each contained 4 L of fermentation medium composed of the pretreated raw cassava starch at a concentration of 200 g/L. Enzyme StargenTM002 and *S. cerevisiae* inoculants (Fali active dry yeast) were added into the fermentation medium at the concentrations of 0.30% (v/w ds) and 0.10% (w/v), respectively. To study the effect of temperature on single-step ethanol production, fermentations were performed at 30 and 40 °C, and without temperature control (with the initial temperature at 40 °C). Fermenters were agitated at the design speed of 200 rpm for 72 h along with the fermentations.

**Fig. 2:**
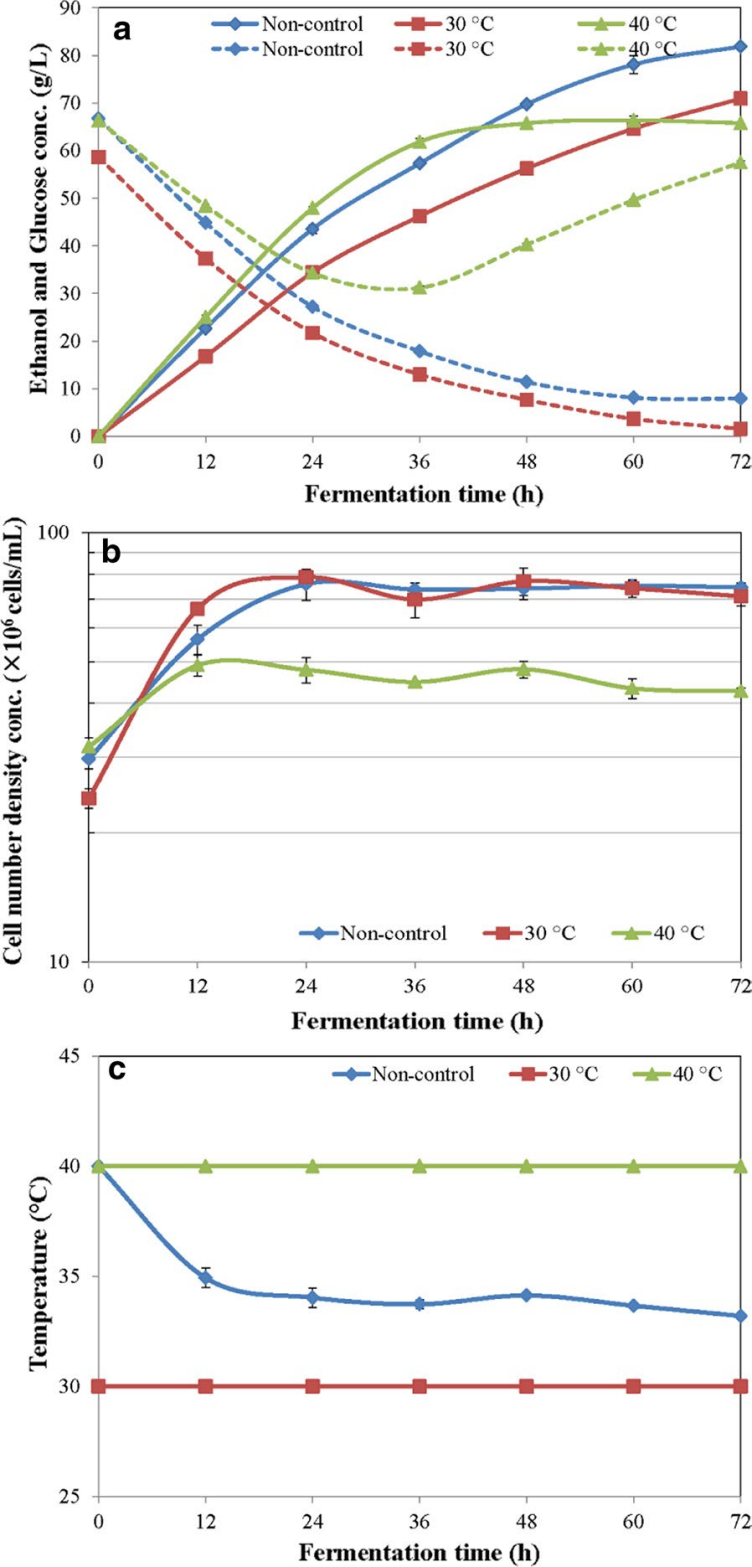
**a** Ethanol production and sugar utilization; **b** cell number density concentration of *S. cerevisiae*; **c** temperature profile, during single-step ethanol fermentations at 30, 40 °C, and a non-control treatment in 5-L fermenters

Temperature is one of the most important factors that affect ethanol production. Figure 2 shows the effect of different temperatures on single-step ethanol production. In Fig. 2a to c, based on the results obtained, it can be observed that the single-step ethanol fermentation at a temperature of 30 °C led to a final ethanol concentration at 70.92 g/L (8.99% v/v). At the non-control temperature (34 ± 1.0 °C) condition, the highest ethanol concentration was obtained at the concentration of 81.86 g/L (10.37% v/v). For the treatment of a temperature increased to 40 °C, it possessed the highest fermentation rate at the first 36 h and after that, it slightly decreased with increasing time. A final ethanol concentration at 65.78 g/L (8.34% v/v) was obtained which was lower than those of the treatments at 30 °C and the non-control temperature. This finding is in a good agreement with [32] who reported that the rate of enzyme catalyzing the reaction in yeast cells increases with temperature up to a certain value and then the enzyme begins to denature resulting in inhibition of yeast activities and consequential decrease in ethanol fermentation. Thus a controlled specific optimum temperature was essentially required for the single-step ethanol production.

The concentration of glucose in fermentation broth at the temperature below 40 °C decreased with time as the fermentation proceeded normally. In contrast to at 40 °C, glucose rapidly decreased within the first 36 h, after that it could not be utilized by yeast which consequently caused the results of final glucose accumulation and lower ethanol in the system.

Although *S. cerevisiae* growth profiles of all fermentation temperatures tended to approach similar values except that at the temperature of 40 °C which was lower than those of the 30 °C and non-control temperatures, results indicate that increasing the fermentation temperature resulted in decreasing the growth and the ethanol production by the yeast. The obtained data clearly show the optimum temperature of around 34 °C at which both yeast and StargenTM002 work best together. If lower than 34 °C, the reaction rate of StargenTM002 declined, and if above this value the yeast cell growth and its activities would be inhibited.

Temperature directly affects metabolism and growth of yeast cells. At a wormer temperature, yeast cells show a rapid decline in viability at the end of fermentation while at an excessively high temperature, enzyme and membrane functions may be disrupted resulting in the stuck fermentation [39]. Moreover, heat stress causes a change of plasma membrane which reduces levels of plasma membrane H^+^–ATPs and transport systems [40]. High temperature showed the inhibitory effect to ethanol production. The intracellular ethanol concentration was higher than the optimum level. Its accumulation within the cells was a consequence of the resistance to its diffusion through cell wall from inside to outside the cells [41]. It affects the plasma membrane of yeast cells resulting in altered membrane organization and permeability [42]. Therefore, during ethanol fermentation, increasing both temperature and ethanol concentration together acts to a reduction in growth rate, fermentation rate, and cell viability. Together both heat and ethanol stress can cause reduction of metabolic activity and eventually cell death.

In this study, we combined the enzymatic hydrolysis of raw cassava starch and ethanol fermentation within a single stage. However, the optimum temperature at 40 °C for the hydrolysis of raw cassava starch was higher than that of the fermentation. The design of operating temperature for single-step ethanol production was very important. As mentioned before, the temperature at 40 °C was optimal for enzyme activity but it could reduce metabolism and growth of *S. cerevisiae* whilst the use of temperature at 30 °C increased the yeast activity, but reduced the hydrolytic rate of raw cassava starch. As explained above, the maximum final ethanol concentration (*p*_max_) at 81.86 g/L (10.37% v/v) with an ethanol yield coefficient (*Y*_*p/s*_) of 0.43 g/g, a productivity (*r*_*p*_) at 1.14 g/L/h, and an efficiency (Ef) of 75.29% was obtained under the non-control fermentation temperature (34 ± 1.0 °C). This result revealed that the single-step ethanol production by *S. cerevisiae* at approximately 34 °C provides the best-compromised temperature that enhanced enzyme activity and promotes *S. cerevisiae* to produce ethanol at a high concentration.

Furthermore, it was interesting to note that by-products, i.e., glycerol, lactic acid, and acetic acid were very low at maximum values of only 1.14, 0.05, and 0.00% (w/v), respectively (Table 4). These major by-products in ethanol production were almost not produced, when using raw cassava starch as the raw material with the single-step fermentation. The advantage of this fermentation system is that the heat pretreatment at 60 °C for 1 h in the early process step reduces acid-producing bacteria contamination resulting in a very low amount of lactic acid and without acetic acid. This method also prevents the system of single-step ethanol fermentation using raw cassava starch from other microorganisms’ contamination.

### Single-step ethanol productions at pilot and industrial scales using the combination of raw cassava starch hydrolysis and fermentation

Major objectives of this section were to (i) evaluate the potential implementation of the single-step ethanol fermentations at the larger scales; (ii) check the ethanol yield and productivity of the single-step fermentations, and (iii) identify problems that were not significantly noticed at the laboratory scale. According to results in laboratory-scale fermentation, further single-step ethanol fermentations were conducted in a 200-L pilot and in a 3000-L industrial fermenter. After enzyme additions and yeast inoculations, single-step ethanol fermentations using the combination of raw cassava starch hydrolysis and fermentation were operated at the same temperature of 34 °C as that of the 5-L fermentation above for 72 h at the agitation speeds of 125 rpm for 200-L fermenter and 55 rpm for 3000-L fermenter. To maintain the designed scale-up parameter, i.e., the energy dissipation rate per unit mass or power input, ${\stackrel{-}{\varepsilon }}_{T}$ of both scales in similarity, both different agitation speeds of the two were consumed with the equivalent power input, ${\stackrel{-}{\varepsilon }}_{T}$ of 0.10 W/kg (Watts/ kg of fermentation broth).

**Fig. 3:**
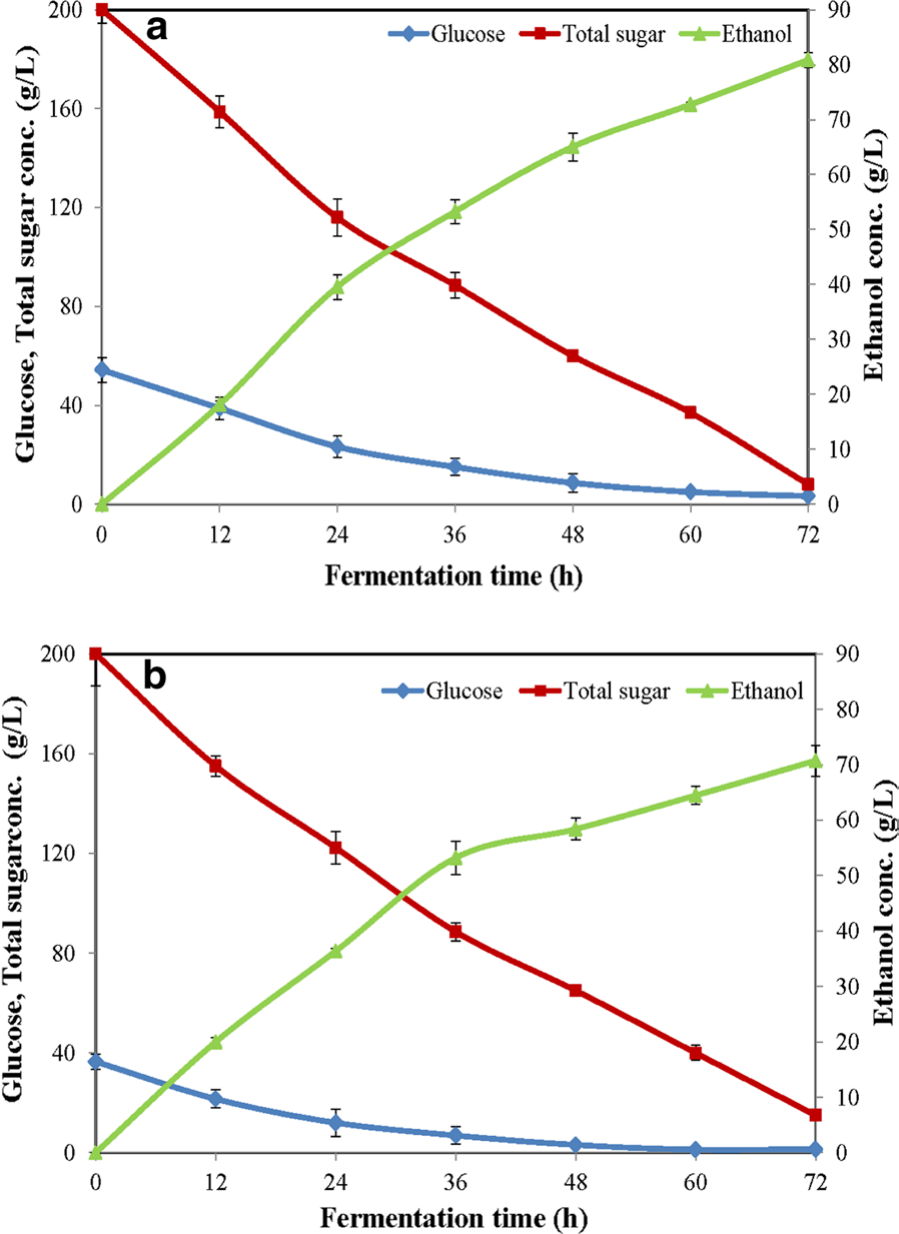
Ethanol productions and substrate utilizations by single-step ethanol fermentations: **a** 200-L pilot-scale fermenter and **b** 3000-L industrial fermenter

The *p*_max_ at 80.90 g/L (10.25% v/v) with a *Y*_*p/s*_ of 0.42 g/g, an *r*_*p*_ at 1.12 g/L/h and an Ef of 74.40% was achieved when the single-step ethanol fermentation from raw cassava starch has been scaled-up to the 200-L fermenter (Fig. 3a). The *p*_max_ of the 200-L fermentation was not significantly different from the *p*_max_ = 81.86 g/L (10.37% v/v) of the 5-L laboratory fermentation. Results in the 200-L fermentation indicated that operations, conditions, and its performances at this scale-up were significantly as effective as those of results obtained in the 5-L fermentation. Furthermore, the single-step ethanol fermentation in the 3000-L industrial fermenter was also studied. In Fig. 3b, it produced the *p*_max_ at 70.74 g/L (8.97% v/v) with a *Y*_*p/s*_ of 0.38 g/g, an *r*_*p*_ at 0.98 g/L/h and, an Ef of 67.56%. Its performances could be observed that there were similar profiles in ethanol production and sugar utilization between both scales of fermentations (Fig. 3a and b).

A comparison of results obtained at different scales of 5-L, 200-L, and 3000-L fermentations is shown in Table 3 and Fig. 3a and b. *p*_max_ values of the 5-L and the 200-L fermenters were very close together with values at 81.86 and 80.90 g/L, respectively, corresponding to *Y*_*p/s*_ of 0.43 and 0.42 g/g, *r*_*p*_ at 1.14 and 1.12 g/L/h, and Ef of 75.29% and 74.40% at both fermentation scales, respectively. They were not significantly different at *p*-value ≤ 0.05. However, the *p*_max_ of the 3000-L fermentation was at 70.74 g/L with a *Y*_*p/s*_ of 0.38 g/g, an *r*_*p*_ at 0.98 g/L/h, and an Ef of 67.56%. Differences of those values, i.e., *p*_max_, *Y*_*p/s*_, *r*_*p*_, and Ef of the 5-L and the 200-L fermentation are around 10% higher than those of the 3000-L fermentation. Reasons why the 3000-L fermentation (Fig. 3b) possessed lower values of *p*_max_, *Y*_*p/s*_, *r*_*p*_, and Ef than those of the 5-L (Fig. 2a) and the 200-L fermentation (Fig. 3a) are as follows. (i) Initial glucose concentrations from starch hydrolyses by StargenTM002 at the start of fermentations were at 66.30, 54.27, and 36.57 g/L for 5-L, 200-L, and 3000-L fermentation, respectively. That of the 3000-L fermenter was the lowest. Differences were due to faster (in 5 and 20 min) temperature controls to reach at 60 °C for raw starch pretreatments at 5-L and 200-L scale fermenter other than the 3000-L fermenter that took a longer time for ~ 30 min. (ii) Final total sugar concentrations left at the end of those fermentations were at 7.90, 8.00, and 15.00 g/L, respectively, affected their ethanol yields. (iii) They consequently, affected rates of substrate utilizations, *r*_*s*_ at values of 2.67, 2.67, and 2.57 g/L/h for 5-L, 200-L, and 3000-L fermentation, respectively.

**Table 3:** Comparison of ethanol concentrations and kinetic parameters of single-step ethanol productions using combination of raw cassava starch hydrolysis and fermentation at different scales of 5-L, 200-L, and 3000-L fermentations by *S. cerevisiae* for 72 h

Fermenters	Ethanol Conc. (g/L)	Ethanol % (v/v)	*Y*_*p/s*_, Yield coefficient (g/g)	Productivity, *r*_*p*_ (g/L/h)	Substrate utilization rate, *r*_*s*_ (g/L/h)	Efficiency (%)
5 L	81.86 ± 1.88^a^	10.37^a^	0.43 ± 0.01^a^	1.14 ± 0.03^a^	2.67 ± 0.14^a^	75.29 ± 1.32^a^
200 L	80.90 ± 0.45^a^	10.25^a^	0.42 ± 0.00^a^	1.12 ± 0.01^a^	2.67 ± 0.12^a^	74.40 ± 0.33^a^
3000 L	70.74 ± 0.56^b^	8.97^b^	0.38 ± 0.00^b^	0.98 ± 0.01^b^	2.57 ± 0.15^b^	67.56 ± 0.39^b^

Statistic comparisons of those mean values within their own columns (among fermentation scales) at *p*-values ≤ 0.05 show different characters, a, b, c, and d, which indicate statically significant differences

Concentrations of major by-products, i.e., glycerol, lactic acid, and acetic acid at the end of single-step ethanol productions remained very low values at every fermentation scale (Table 4). Minimum inhibitory concentrations of lactic and acetic acid were at 0.80% and 0.05%, respectively [30]. In this research work, concentrations of both acid by-products remained lower than those of minimum stressful values.

**Table 4:** Concentrations of by-products of glycerol, lactic acid, and acetic acid from single-step ethanol productions using combination of raw cassava starch hydrolysis and fermentation at different scales of 5-L, 200-L, and 3000-L fermentations by *S. cerevisiae* for 72 h

Fermentations	Glycerol (g/L)	Lactic acid (g/L)	Acetic acid (g/L)
5-L fermenter	11.39 ± 0.11	0.46 ± 0.00	0.00 ± 0.00
200-L fermenter	11.02 ± 0.03	0.51 ± 0.01	0.23 ± 0.01
3000-L fermenter	9.39 ± 0.07	0.52 ± 0.01	0.59 ± 0.00

No statistic comparisons of those mean values within their own columns (among fermentation scales) because those values are very low and less than those of the values to inhibit yeast cell growth and affect ethanol yield

It is clearly shown that at 200-L and 3000-L scales of fermentations, values of ethanol content, glucose left, glycerol, lactic acid, and acetic acid during single-step ethanol productions using the combination of raw cassava starch hydrolysis and fermentation were similar to those values obtained at the 5-L laboratory scale. This indicated that there were no deviations of those results, i.e., *p*_max_, *Y*_*p/s*_, *r*_*p*_, and Ef including minimal by-products obtained when the 5-L fermentation was scaled-up to the pilot scale, and further to the industrial scale of operations with around 10% deviation, where is statistically acceptable. This proves and supports the scalable potential and feasibility for the decision to the new route for the industrial bioethanol production from raw starch.

### Advantages

Advantages of this research work are as follows: (i) the production of ethanol from raw starch can be done in a single-step of operation without liquefaction at very high temperature, and saccharification. (ii) Consequently, complexities, times, and costs, of operations can significantly be minimized. (iii) As the raw starch is used as the substrate, major by-products, i.e., glycerol, lactic acid, and acetic acid are essentially minimized. (iv) With high concentrations, yields, productivities, and efficiencies of ethanol productions at all experimental scales, comparable with those in commercial industries that use molasses and hydrolyzed starch as raw materials, this can be implemented in the industries. (v) Using fluid dynamics for the design of impellers speeds and operations, the lysis reactor for raw starch pretreatment, and the ethanol fermenter at every scale can be designed and scaled-up to the industries.

## Conclusions

The combination of raw cassava starch hydrolysis and fermentation is practicable for the single-step ethanol production. The granular starch hydrolyzing enzyme StargenTM002 showed a high ability for raw cassava starch hydrolysis. Under the optimum condition, 68.56% (w/w) of raw cassava starch was converted to glucose. Moreover, the pretreatment of raw cassava starch at 60 °C for 1 h with Distillase ASP (a blend of glucoamylase and pullulanase) greatly improved subsequent further raw starch hydrolysis by StargenTM002 which converted 80.19% (w/w) of raw cassava starch to glucose. For ethanol production from raw cassava starch, the highest ethanol concentration from single-step fermentation by *S. cerevisiae* (Fali, active dry yeast) at 34 °C in a 5-L fermenter was achieved. This ethanol was produced at the concentration of 81.86 g/L (10.37% v/v) with a yield coefficient of 0.43 g/g, a productivity at 1.14 g/L/h, and an efficiency of 75.29%. The scale-up from the 5-L laboratory fermenter to the 200-L pilot-scale, and further to the 3000-L industrial fermenter was essentially successful. There were no significant differences of those values, results, and performances between the 5-L and the 200-L fermentation scales. There were little differences of those values of the 3000-L scale comparable with the former two. These results indicated that the single-step ethanol production using the combination of raw cassava starch hydrolysis and fermentation can be scaled-up to the novel industrial production of bioethanol.

## Materials

### Microorganism

The manufactured active dry yeast, *Saccharomyces cerevisiae* (Fali), obtained from AB Mauri (Australia), was used in this study. This yeast strain could produce the maximum ethanol yield exceeding 18% (v/v) or 12% (w/v) depending on fermentation procedures. Furthermore, it is extremely thermotolerant and it has a wide range of fermentation temperatures from 25 to 40 °C. The active dry yeast inoculants were re-hydrated in distilled water at 40 °C for 20 min prior to inoculations into the single-step ethanol fermentations.

### Materials

Cassava starch of the “Three Elephants” brand was obtained from Chorchiwat Industry Co., Ltd (Chon Buri province, Thailand) with complements. After manufacture, freshly cassava starch was kept at a dry and cool place in the laboratory store. Moisture content and other compositions of the cassava starch in this study were totally ~ 10 ± 1.00 (% w/w) so that the carbohydrate (fermentable carbon source) is ~ 90 ± 1.00 (% w/w). Its pH was 5–7. Cassava starch chemical compositions, i.e., carbohydrate, moisture, crude fiber, ash, protein, and fat were 90.80 ± 1.22, 7.10 ± 0.10, 1.20 ± 0.00, 0.45 ± 0.22, 0.32 ± 0.01, and 0.17 ± 0.00 (% w/w), respectively [43].

Commercial enzymes provided by DuPont Industrial Biosciences (previously known as Genencor, A Danisco Division) were utilized in this study. They were (i) StargenTM002 (granular starch hydrolyzing enzyme, containing *Aspergillus kawachi* α-amylase expressed in *Trichoderma reesei* and glucoamylase from *T. reesei*) and (ii) Distillase ASP (containing a blend of glucoamylase from *Bacillus licheniformis* and bacterial pullulanase from *T. reesei*). Properties of these commercial enzymes are presented in Table 5.

**Table 5:** Characterizations of the commercial enzymes used in this study

Commercial enzymes (types)	Optimum temperature (°C)	Optimum pH	Activity
StargenTM002 (blend of alpha-amylase and glucoamylase)	20–40	4.0–4.5	570 GAU/g
Distillase ASP (blend of glucoamylase and pullulanase)	58–65	4.0–4.5	580 GAU/g

GAU/g means glucoamylase unit (GAU). One GAU unit, defined by DuPont, is the amount of enzyme that will liberate 1 g of reducing sugars calculated as glucose per hour from soluble starch substrate under the assay conditions

## Methods

### Optimization of StargenTM002 conditions for raw cassava starch hydrolysis

Volumes of 1-L starch slurry in glass containers, each containing 20% (w/v) of raw cassava starch prepared in distilled water, were incubated in a water bath at 30 °C for 48 h, with continuous stirring at 100 rpm using an overhead stirrer. StargenTM002 was added into raw cassava starch slurries at concentrations of 0.10–0.40% (v/w of dry starch basis, ds) before starts of hydrolysis.

For the study of temperature effect on raw cassava starch hydrolysis, starch slurries were incubated at 30–40 °C with StargenTM002 at a concentration of 0.10% (v/w ds). Effects of pH on raw cassava starch hydrolysis were verified from 3.0 to 7.0. Starch slurries at pH 3.0–4.0 were prepared in sodium acetate buffer and those at pH 5.0–7.0 were prepared in potassium phosphate buffer before hydrolyses by StargenTM002 at a concentration of 0.10% (v/w ds).

### Raw cassava starch hydrolysis in 15-L lysis reactor

A 10 L of starch slurry, containing 20% (w/v) of raw cassava starch prepared in sodium acetate buffer at pH 4.0, was hydrolyzed by StargenTM002 at a concentration of 0.30% (v/w ds) in a 15-L lysis reactor at 40 °C and agitated at 220 rpm (equivalent to the power input, ${\stackrel{-}{\varepsilon }}_{T}$ of 0.10 W/kg) for 72 h. During hydrolysis, samples of 10 mL were withdrawn at every 12-h intervals for analyses. The pH value of each sample was adjusted to 1.5–1.6 with 2 M HCl solution to stop enzyme activities [14].

### Raw cassava starch pretreatments and subsequent hydrolysis in 15-L lysis reactor

The heat pretreatment of cassava starch at its below gelatinization temperature before being subjected to enzyme hydrolysis can increase the degree of conversion (hydrolysis) of native starch to release free glucose molecules [14]. In addition, it was also reported that the combination of urea addition and pre-heating treatment at a sub-gelatinization temperature greatly facilitated the hydrolysis by StargenTM002 [44]. Therefore, in this study, the 10 L of starch slurries, containing 20% (w/v) of raw cassava starch prepared in sodium acetate buffer at pH 4.0, were pretreated with heating at 60 °C in the 15-L lysis reactors (in-house design and fabrication by our group, fit with 2-Ekato Intermig, the high-efficiency impellers (Germany) of the diameter, *D* of 0.115 m, agitating at 220 rpm (${\stackrel{-}{\varepsilon }}_{T}$= 0.10 W/kg) for 1 h. The 10 L of raw starch slurries above were pretreated with the additions of (i) Distillase ASP at the concentrations of 0.10–0.30% (v/w ds) and (ii) urea at the concentrations of 1.0–3.0% (v/w ds). After the pretreatments, the raw starch slurries were hydrolyzed by StargenTM002 at the concentration of 0.30% (v/w ds) at 40 °C and stirring at the same speed of 220 rpm with ${\stackrel{-}{\varepsilon }}_{T}$= 0.10 W/kg for 72 h.

### Single-step ethanol production using combination of raw cassava starch hydrolysis and fermentation at different temperatures in 5-L fermenter

In order to study the effect of temperatures on single-step ethanol production, fermentations were performed at different temperatures of 30, 40 °C, and without temperature control (with the initial temperature of 40 °C). pH values were not controlled, but undergoing with the initial value at 5.5. They were carried out using 5-L fermenters (Biostat B, B. Braun Biotech International, Germany), each fit with 2 Rushton turbine impellers of the diameter, D of 0.065 m, operating at the designed agitation speed of 200 rpm, equivalent to the power input, ${\stackrel{-}{\varepsilon }}_{T}$ of 0.10 W/kg, for 72 h. 5-L fermenters were contained with 4 L of fermentation medium which was composed of 200 g/L (18% w/v carbohydrate, based on deduction of 10% (w/v) of moisture and other contents) of the pretreated raw cassava starch slurry plus 40 g/L (4% w/v) of sugarcane molasses containing 50% (w/v) of sucrose to meet the total carbon source concentration of 20% (w/v). Inorganic salts, i.e., (NH_4_)_2_HPO_4_, HK_2_PO_4_, Na_2_HPO_4_, and MgSO_4_.7H_2_O, were also supplemented into the medium at concentrations of 0.1, 1.5, 1.8, and 3.8 g/L, respectively. After homogeneous medium mixing, StargenTM002 was added at a concentration of 0.30% (v/w ds) and inoculants of re-hydrated active dry yeast, *S. cerevisiae* Fali, were further inoculated into fermenters at a final concentration of 1.0 g/L (0.10% w/v). During fermentations, samples of 10 mL were withdrawn at every 12 h interval for analyses.

### Single-step ethanol production using combination of raw cassava starch hydrolysis and fermentation at 200-L pilot and 3000-L industrial scales

(1) The pilot-scale ethanol fermentation was carefully conducted using the 200-L fermenter of 0.50 m diameter, *T* and 1.00 m height, *H* (in-house design and fabrication by our group, fit with 2 Ekato Intermig impellers of the diameter, D of 0.30 m, agitating at 125 rpm (equivalent to the power input, ${\stackrel{-}{\varepsilon }}_{T}$ of 0.10 W/kg). The fermenter was contained with 150 L of the fermentation medium^p^.

(2) The industrial ethanol fermentation was practically implemented using the 3000-L fermenter of 1.25 m diameter, T and 2.50 m height, H (industrial design by our group and fabrication by Chorchiwat Industry Co., Ltd. (CCW)), fit with 2 Ekato Intermig impellers of the diameter, D of 0.85 m, agitating at 55 rpm (equivalent to the power input, ${\stackrel{-}{\varepsilon }}_{T}$ of 0.10 W/kg). The fermenter was contained with 2100 L of the fermentation medium^I^.

The fermentation medium^P^ and medium^I^ of both scales were definitely the same in compositions and concentrations. Like the 5-L medium composition above, they were composed of 20% (w/v) of the pretreated raw cassava starch slurry plus 4.0% (w/v) of sugarcane molasses containing 50% (w/v) sucrose, and 0.10 g/L (NH_4_)_2_HPO_4_, 1.50 g/L of HK_2_PO_4_, 1.80 g/L of Na_2_HPO_4_, and 3.80 g/L of MgSO_4_.7H_2_O. When mixed well and reached the designed set-point temperature at 34 °C and the initial pH at 5.5 (after 24 h, pH declined to 4.5 constant) the enzyme StargenTM002 was added into the fermenters at a concentration of 0.30% (v/w ds) and subsequently inoculants of re-hydrated active dry yeast, *S. cerevisiae* Fali, were inoculated at a concentration of 0.10% (w/v). Fermentations were performed at the temperature of 34 ± 1.0 °C for 72 h at designed agitation speeds of each fermenter mentioned above. During fermentations, samples of 10 mL were withdrawn at every 12-h interval for further analyses.

Studies of single-step ethanol productions at the 200-L pilot-scale and the 3000-L industrial-scale fermenters were practically based on principles of scale-up rules: (i) geometric similarity where dimensional lengths of all fermenter geometries among different fermenter scales were designed to maintain the same values; (ii) conditional similarity where all optimal conditions, i.e., temperature, pH, substrate and enzyme concentrations were imitated from the 5-L laboratory research work; (iii) operational similarity where a selected key operational method was designed. For this work, the power input (energy dissipation rate per unit mass or power input, $\it {\stackrel{-}{\upvarepsilon }}_{\text{T}}$) was maintained similar (kept constant) with the value of ${\stackrel{-}{\varepsilon }}_{T}$ = 0.10 (W/kg), thus impeller agitation speeds among different scales were empirically calculated (see details below).

## Analyses and quantitative methods

### Analytical method using HPLC to quantify concentrations of glucose, ethanol, and by-products

The high-performance liquid chromatography (HPLC) method was utilized to quantify concentrations of glucose, ethanol, glycerol, lactic acid, and acetic acid in fermentation broth samples by comparisons with standards of those known concentration values. The HPLC system (KNAUER Smartline, Berlin, Germany) with the refractive index (RI) detector (Smartline 2300) and with the Eurokat H vertex column was used. An eluent solution of 0.01 N H_2_SO_4_ was utilized at a flow rate of 0.8 mL/min. Analyses were performed at 60 °C. Samples were diluted 10 times, filtered through 0.45-µm filters, and injected into the column with an amount of 20 µL [27, 45]*.*

### Total sugar analysis

Total sugars mean all concentrations of carbon sources, i.e., raw starch plus free residual glucose of which raw starch in the mixture of fermentation broth was completely hydrolyzed by acid to release all glucose molecules, and then glucose from both sources in the same mixture was measured as the total sugar or total glucose. The total sugar of cultured samples during fermentation was analyzed by the modified sulfuric acid hydrolysis method. Samples of 1 mL in micro-tubes were centrifuged at 10× rpm for 10 min. Supernatants of 0.5 mL were transferred into 20-mL test tubes and then 2-mL volumes of 2 N H_2_SO_4_ solution were added, mixed well, and capped. Test tubes with mixtures were boiled in a water bath at 95 °C for 30 min. Neutralizations were done with the addition of 4 N NaOH and re-centrifugations were operated to precipitate residues. Supernatants were analyzed using the HPLC to quantify concentrations of glucose as the total sugar [27, 45]*.*

### Scanning electron microscopy

Photographic characteristics of the pretreated and hydrolyzed raw cassava starch granules were observed under the SEM (scanning electron microscope, LEO, 1450VP, Germany). Samples were mounted on circular aluminum stubs with carbon tape, coated with gold, and examined in SEM at an accelerating voltage of 10 kV and photographed.

### Kinetic parameters’ calculations

#### Starch hydrolyses

The degree of conversion of raw starch to glucose was calculated as the percentage of glucose released from the raw cassava starch hydrolysis using the equation:

1 $$Degree\,of\,conversion\, \left(\%\right)= \frac{Glucose \, released (\text{g}/\text{L})}{Raw\, cassava\, starch\, used (\text{g}/\text{L})\times 1.11 (\text{g}/\text{g})} 100,$$

where 1.11 (g/g) is the 1.11 g theoretical (stoichiometric) yield of glucose from 1.00 g of starch, calculated from $\frac{\text{n}\left({\text{C}}_{6}{\text{H}}_{12}{\text{O}}_{6}\right)}{{\left({\text{C}}_{6}{\text{H}}_{12}{\text{O}}_{6}\right)}_{\text{n}}-((\text{n}-1)\times {\text{H}}_{2}\text{O})}$, where C_6_H_12_O_6_ is the glucose with a molecular weight (MW) of 180.156 g/mol; H_2_O is the water with a MW of 18.015 g/mol, and n is the number of glucose molecules of glucose and of starch or degree of polymerization (Dp). For example, if a starch chain polymer of 1000 glucose molecules was completely hydrolyzed, where n = 1000 molecules, then substitute into the above term to obtain $\frac{1000\left(180.156\right)}{{((180.156)}_{\text{1,0}00})-(\left(1000-1\right)\times 18.015)}=$ 1.11 g/g. Although n or Dp values vary, the number of 1.11 g/g is still obtained as a constant (see Additional file 2).

#### Ethanol productions

Three kinetic parameters of ethanol productions were calculated using experimental data, i.e., cultivation time, t (h), concentration of ethanol, *p* (g/L), and concentration of raw starch carbon substrate used, *s* (g/L). (i) The yield coefficient of ethanol production, *Y*_*p/s*_ (g/g) is from *Y*_*p/s*_$=\boldsymbol{ }\frac{\Delta p}{\Delta s}$, where Δ*p* is the ethanol produced (g/L) and Δ*s* is the substrate used (g/L), (ii) The productivity or production rate of ethanol, *r*_*p*_ (g/L/h) is from ${r}_{p}=\frac{dp}{dt}$. (iii) The utilization rate of substrate, *r*_*s*_ (g/L/h) is from ${r}_{s}=\frac{ds}{dt}$. The production efficiency, Ef (%) is from Ef$\boldsymbol{ }=\frac{{Y}_{p/s}}{{Y^{\prime}}_{p/s}}100$, where *Y*_*p/s*_ is from the experiment (observed value) and *Y'*_*p/s*_ is from the theoretical yield coefficient or stoichiometry [45]. The theoretical yield, Y'_p/s_ of ethanol from starch is 0.566 g/g, calculated from${Y^{\prime}_{p/s}}=\frac{{1.11}^{a}\times {0.51}^{b}}{1.0}$, where *“a”* is the glucose yield of 1.11 g from 1.00 g of starch as in Eq. (1) that has been profoundly explained above, and “b” is the theoretical yield (Y'_p/s_) of ethanol from 1.00 g of glucose, calculated from ${Y^{\prime}_{p/s}}=\frac{2(46.07)}{180.156}=0.51$ g/g, where molecular weights of ethanol and glucose are 46.07 and 180.156 g/mol, respectively, and one molecule of glucose stoichiometrically produces two molecules of ethanol.

### Calculations of designed fermenter impeller speeds

For fluid dynamics in the stirred tank bioreactor [46, 47], the power, *P* (W) is calculated from $P=nPo\rho {N}^{3}{D}^{5}$, where *n* is the number of impellers of 2 for all the 5-L fermenter, 15-L lysis reactor, 200 pilot-scale fermenter, and 3000-L industrial fermenter. Po is the power number of 5 (no unit or dimensionless) for one Rushton turbine impeller of the 5-L fermenter and *Po *of 0.33 for one Ekato Intermig (high-efficiency) impeller of every scale, i.e., 15-L lysis reactor, 200-L and 3000-L fermenters. $\rho$ is the fluid density (kg/m^3^) of lysis reactor or fermenter. *N* is the impeller speed (rps), and *D* is the impeller diameter, where *D* of 0.065 m is for the 5-L fermenter, 0.115 m is for the 15-L lysis reactor, 0.30 m is for the 200-L fermenter, and 0.85 m is for the 3000-L fermenter.

The power input or energy dissipation rate per unit mass, $\it {\stackrel{-}{\varepsilon }}_{\text{T}}$ (W/kg) is calculated from

2 $$~\bar{\varepsilon }_{T} \;{\text{or}}\;\frac{P}{{\rho V}} = \frac{{nPo\rho N^{3} D^{5} }}{{\rho V}},$$

where *V* is the fluid volume (m^3^). Thus, designed impeller speeds, *N*, of the power input of 0.10 (W/kg) are calculated from

3 $$N = ((\bar{\varepsilon }_{T} V)/(nPoD^{5} ))^{{(1/3)}} .$$

Both these kinetic and fluid dynamic parameters from the laboratory and the pilot-scale experiment are very crucial for the scale-up of further fermentation at the industrial scale. That is the foreseen reason why impeller speeds were designed based on the power input rather than just the speed in rpm (Table 6).

**Table 6:** Lysis reactor and fermenter designs, and scale-up values for this research work; they were designed by maintaining geometric similarities and the same power input, ${\stackrel{-}{\varepsilon }}_{T}$ among different fermenter scales constant with the value of 0.10 W/kg

Reactor types; total vol. (L)	Working vol. (L)	Dimension *H/T* (m/m) Height/tank Ø	Impeller type	No.	Impeller diameter, *D* (m)	Speed (rpm)	Power input, ${\stackrel{-}{\varepsilon }}_{T}$ (W/kg)
15-L lysis reactor	10	0.40/0.20	Ekato Intermig	2	0.115	220	0.10
5-L fermenter	4	0.35/0.15	Rushton Turbine	2	0.065	200	0.10
200-L fermenter	150	1.00/0.50	Ekato Intermig	2	0.300	125	0.10
3000-L fermenter	2100	3.00/1.50	Ekato Intermig	2	0.850	55	0.10

### Statistical analysis

Statistical analyses of each data set from each experiment were undertaken with the one-way analysis of variance (ANOVA). Differences of treatment mean values from three replications (each experiment was done in three replicates) were compared with the Tukey’s range test method at *p*-value ≤ 0.05 using the Minitab version 17 software. Standard errors (± SE) were shown together with mean values as error bars in the graph. If the error bars of SE in the plots did not  appear, assuming that they were smaller than the sizes of legends.

## Supplementary Information

**Additional file 1.**

**Additional file 2.**

## Data Availability

Supplementary materials: (I) https://www.researchgate.net/publication/345716528—Details show yields of different crops per unit area, starch or sugar contents in each crop, theoretical yield coefficients, *Y’**p/s* (mass of ethanol/mass of starch or sugar) produced stoichiometrically, and calculated yields of ethanol per unit area. (II) https://www.researchgate.net/publication/345716731—Degree of starch conversion, hydrolysis of starch to release glucose (molecules) and yield coefficient.

## Abbreviations

BL: Billion liters
1G: First generation feedstock
2G: Second generation feedstock
ha: Hectare
MT: Metric ton
CBP: Consolidated bioprocessing
CP: Cassava pulp
*p*_max_: Maximum product
*Y*_*p/s*_: Observed product yield coefficient
*Y*'_*p/s*_: Theoretical yield coefficient
*r*_*p*_: Productivity or production rate
*r*_*s*_: Substrate utilization rate
Ef: Efficiency
SSF: Simultaneous saccharification and fermentation
w/w: Weight by weight
v/v: Volume by volume
v/w ds: Volume by weight of dry solid
g/L/h: Gram per liter per hour
L: Liter
m: Meter
H^+^–ATP: Hydrogen ion–adenosine triphosphate
g/g: Gram per gram
g/L: Gram per liter
${\stackrel{-}{\varepsilon }}_{T}$: Power input or energy dissipation rate per unit mass
W/kg: Watts per kilogram
GAU: Glucoamylase unit
rpm: Revolutions per minute
rps: Revolutions per second
M HCl: Molar of hydrochloric acid conc.
(NH_4_)_2_HPO_4_: Diammonium hydrogen phosphate
HK_2_PO_4_: Hydrogen dipotassium phosphate
Na_2_HPO_4_: Disodium hydrogen phosphate
MgSO_4_.7H_2_O: Magnesium sulfate
N H_2_SO_4_: Normal of sulfuric acid conc.
N NaOH: Normal of sodium hydroxide conc.
*H*: Height
*T*: Tank diameter
*D*: Impeller diameter
CCW: Chorchiwat Industry Co. Ltd.
HPLC: High-performance liquid chromatography
µm: Micrometer
µL: Microliter
SEM: Scanning electron microscope
*p*: Conc. of ethanol product
*t*: Time
*s*: Carbon substrate (glucose or starch)
Δ*p*: Ethanol produced
Δ*s*: Substrate used
*d*: Differential
*P*: Power
*n*: Number of impellers
*Po*: Impeller power number
*N*: Impeller speed in rps
*ρ*: Fuid density
*V*: Volume
Ø: Diameter
ANOVA: Analysis of variance
SE: Standard error

## References

[1] Cripwell RA, Favaro L, Viljoen-Bloom M, van Zyl WH (2020). Consolidated bioprocessing of raw starch to ethanol by *Saccharomyces cerevisiae*: achievements and challenges. Biotechnol Adv.

[2] Siqueira PF, Karp SG, Carvalho JC, Sturm W, Rodríguez-León JA, Tholozan J, Singhania RR, Pandey A, Soccol CR (2008). Production of bio-ethanol from soybean molasses by *Saccharomyces cerevisiae* at laboratory, pilot and industrial scales. Biores Technol.

[3] Benjaphokee S, Hasegawa D, Yokota D, Asvarak T, Auesukaree C, Sugiyama M, Kaneko Y, Boonchird C, Harashima S (2012). Highly efficient bioethanol production by a *Saccharomyces cerevisiae* strain with multiple stress tolerance to high temperature, acid and ethanol. New Biotechnol.

[4] Hashem M, Zohri ANA, Ali MMA (2013). Optimization of the fermentation conditions for ethanol production by new thermotolerant yeast strains of *Kluyveromyces* sp.. Afr J Microbiol Res.

[5] Renewable Fuels Association (RFA), 2020. Focus Forward: 2020 Pocket Guide to Ethanol. https://ethanolrfa.org/wp-content/uploads/2020/02/2020-Outlook-Pocket-Guidefor-Web.pdf, Accessed 4 Nov 2020.

[6] Sorapipatana C, Yoosin S (2011). Life cycle cost of ethanol production from cassava in Thailand. Renew Sustain Energy Rev.

[7] Naguleswaran S, Li J, Vasanthan T, Bressler D, Hoover R (2012). Amylolysis of large and small granules of native triticale, wheat, and corn starches using a mixture of α-amylase and glucoamylase. Carbohyd Polym.

[8] Ogbonna CN, Okoli EC (2010). Conversion of cassava flour to fuel ethanol by sequential solid state and submerged cultures. Process Biochem.

[9] Cripwell RA, Rose SH, Favaro L, van Zyl WH (2019). Construction of industrial *Saccharomyces cerevisiae* strains for the efficient consolidated bioprocessing of raw starch. Biotechnol Biofuels.

[10] Sakwa L, Cripwell RA, Rose SH, Viljoen-Bloom M (2018). Consolidated bioprocessing of raw starch with *Saccharomyces cerevisiae* strains expressing fungal alpha-amylase and glucoamylase combinations. FEMS Yeast Res.

[11] Shigechi H, Fujita Y, Koh J, Ueda M, Fukuda H, Kondo A (2004). Energy-saving direct ethanol production from low-temperature-cooked corn starch using a cell-surface engineered yeast strain co-displaying glucoamylase and α-amylase. Biochem Eng J.

[12] Azmi AS, Ngoh GC, Mel M, Hasan M, Lima MAP, Natalense APP (2012). Single-step bioconversion of unhydrolyzed cassava starch in the production of bioethanol and its value-added products. Bioethanol.

[13] Matías J, Encinar JM, González J, González JF (2015). Optimization of ethanol fermentation of Jerusalem artichoke tuber juice using simple technology for a decentralized and sustainable ethanol production. Energy Sustain Dev.

[14] Shariffa YN, Karim AA, Fazilah A, Zaidul ISM (2009). Enzymatic hydrolysis of granular native and mildly heat-treated tapioca and sweet potato starches at sub-gelatinization temperature. Food Hydrocolloids.

[15] Gohel V, Duan G (2012). No-cooked process for ethanol production using Indian broken rice and pearl millet. Int J Microbiol..

[16] Nguyen CN, Le TM, Chu-Ky S (2014). Pilot scale simultaneous saccharification and fermentation at very high gravity of cassava flour for ethanol production. Ind Crops Prod.

[17] Cha YL, An GH, Yang J, Moon YH, Yu GD, Ahn JW (2015). Bioethanol production from *Miscanthus* using thermotolerant *Saccharomyces cerevisiae* mbc 2 isolated from the respiration-deficient mutants. Renew Energy.

[18] Lamsal BP, Wang H, Johnson LA (2011). Effect of corn preparation methods on dry-grind ethanol production by granular starch hydrolysis and partitioning of spent beer solids. Biores Technol.

[19] Zhang P, Chen C, Shen Y, Ding T, Ma D, Hua Z, Sun D (2013). Starch saccharification and fermentation of uncooked sweet potato roots for fuel ethanol production. Biores Technol.

[20] Xu QH, Yan YS, Feng JX (2016). Efficient hydrolysis of raw starch and ethanol fermentation: a novel raw starch-digesting glucoamylase from *Penicillium oxalicu*. Biotechnol Biofuels.

[21] Brown A, Waldheim L, Landalv I, Saddler J, Ebadian M, McMillan JD, Bonomi A, Klein B. Advanced Biofuels–Potential for Cost Reduction. Published by IEA Bioenergy. 2020; https://www.ieabioenergy. com/wp-content/uploads/2020/02/T41_CostReductionBiofuels-11_02_19-final.pdf

[22] Roble ND, Ogbonna J, Tanaka H (2020). Simultaneous amylase production, raw cassava starch hydrolysis and ethanol production by immobilized *Aspergillus awamori* and *Saccharomyces cerevisiae* in a novel alternating liquid phase–air phase system. Process Biochem.

[23] Yeunyaw P, Yuwa-amornpitak T (2018). Bioconversion of cassava starch to bio-ethanol in a single step by co-cultures of *Amylomyces rouxii* and *Saccharomyces cerevisiase*. Songklanakarin J Sci Technol.

[24] Myburgh MW, Rose SH, Viljoen-Bloom M (2020). Evaluating and engineering *Saccharomyces cerevisiae* promoters for increased amylase expression and bioethanol production from raw starch. FEMS Yeast Res..

[25] Trakarnpaiboon S, Srisuk N, Piyachomkwan K, Sakai K, Kitpreechavanich V (2017). Enhanced production of raw starch degrading enzyme using agro-industrial waste mixtures by thermotolerant *Rhizopus microsporus* for raw cassava chip saccharification in ethanol production. Prep Biochem Biotech.

[26] Siriwong T, Laimeheriwa B, Aini UN, Cahyanto MN, Reungsang A, Salakkam A (2019). Cold hydrolysis of cassava pulp and its use in simultaneous saccharification and fermentation (SSF) process for ethanol fermentation. J Biotechnol.

[27] Malairuang K, Krajang M, Rotsattarat R, Chamsart S (2020). Intensive multiple sequential batch simultaneous saccharification and cultivation of *Kluyveromyces marxianus*SS106 thermotolerant yeast strain for single-step ethanol fermentation from raw cassava starch. Processes Spec Issue Adv Microb Ferment Process..

[28] Yalcin SK, Ozbas ZY (2008). Effect of pH and temperature on growth and glycerol production kinetics of two indigenous wine strains of *Saccharomyces cerevisiae* from Turkey. Brazil J Microbiol.

[29] Liu X, Jia B, Sun X, Ai J, Wang L, Wang C, Zhao F, Zhan J, Huang W (2015). Effect of initial pH on growth characteristics and fermentation properties of *Saccharomyces cerevisiae*. J Food Sci.

[30] Breisha GZ (2010). Production of 16% ethanol from 35% sucrose. Biomass Bioenerg.

[31] Bai FW, Anderson WA, Moo-Young M (2008). Ethanol fermentation technologies from sugar and starch feed stocks. Biotechnol Adv.

[32] Wong YC, Sanggari V (2014). Bioethanol production from sugarcane bagasse using fermentation process. Orient J Chem.

[33] Arroyo-Lopez FN, Orlic S, Querol A, Barrio E (2009). Effect of temperature, pH, and sugar concentration on the growth parameters of *Saccharomyces cerevisiae*, *S. kudriavzevii* and their interspecific hybrid. Int J Food Microbiol.

[34] Uthumporn U, Zaidul ISM, Karim AA (2010). Hydrolysis of granular starch at sub-gelatinization temperature using a mixture of fungal amylolytic enzymes. Food Bioprod Process.

[35] Chen Y, Huang S, Tang Z, Chen X, Zhang Z (2011). Structural changes of cassava starch granules hydrolyzed by a mixture of α-amylase and glucoamylase. Carbohyd Polym.

[36] Nadir N, Mel M, Karim MIA, Yunus RM (2009). Comparison of sweet sorghum and cassava for ethanol production by using *Saccharomyces cerevisiae*. J Appl Sci.

[37] Chu-Ky S, Pham TH, Bui KLT, Nguyen TT, Pham KD, Nguyen HDT, Luong HN, Tu VP, Nguyen TH, Ho PH, Le TM (2016). Simultaneous liquefaction, saccharification and fermentation at very high gravity of rice at pilot scale for potable ethanol production and distillers dried grains composition. Food Bioprod Process.

[38] Tawil G, Nielsen AV, Sabate AR, Colonna P, Buleon A (2012). Hydrolysis of concentrated raw starch: a new very efficient α-amylase from *Anoxybacillus flavothermus*. Carbohyd Polym.

[39] Sener A, Canbas A, Unal MU (2007). The effect of fermentation temperature on the growth kinetics of wine yeast species. Turk J Agric For.

[40] Jimoh SO, Ado SA, Ameh JB, Whong CMZ (2013). Heat-shock and ethanol-osmotic effect on fermentable yeast cells. Int J Biol Sci.

[41] Navarro JM, Durand G (1978). Alcohol fermentation: effect of temperature on ethanol accumulation within yeast cells. Ann Microbiol.

[42] Quintas C, Lima-Costa E, Loureiro-Dias MC (2000). The effect of ethanol on the plasma membrane permeability of spoilage yeasts. Food Technol Biotechnol.

[43] Chinma CE, Ariahu CC, Abu JO (2013). Chemical composition, functional and pasting properties of cassava starch and soy protein concentrate blends. J Food Sci Technol.

[44] Li J, Vasanthan T, Bressler DC (2012). Improved cold starch hydrolysis with urea addition and heat treatment at subgelatinization temperature. Carbohyd Polym.

[45] Malairuang K, Krajang M, Jatuporn S, Rattanapradit K, Chamsart S (2020). High cell density cultivation of *Saccharomyces cerevisiae* with intensive multiple sequential batches together with a novel technique of fed-batch at cell level (FBC). Processes Spec Issue Adv Microb Ferment Process..

[46] Chamsart S (2001). A cell lysis reactor for the production of plasmid DNA from recombinant *E. coli* for gene therapy.

[47] Nienow AW (1997). Bioreactor and bioprocess fluid dynamics (British Hydromechanics Research Group (REP)).

