# Supplementary material for: Single-step ethanol production from raw cassava starch using a combination of raw starch hydrolysis and fermentation, scale-up from 5-L laboratory and 200-L pilot plant to 3000-L industrial fermenters
Source: Biotechnol Biofuels. 2021 Mar 16;14:68. doi: 10.1186/s13068-021-01903-3 (PMC7962325; doi:10.1186/s13068-021-01903-3)
Supplement: Supplementary file 1 — Additional file 1. [file 13068_2021_1903_MOESM1_ESM.pdf]

Supplementary Material I

Yield of Ethanol per Unit Area of Cultivation

Starch and sugar are the first generation (1G) feedstocks and the most abundant renewable carbon sources and are more readily digestible for conversions to biofuels than cellulosic feedstocks which are the second generation (2G) raw material. Cultivations of different crops gain different quantities of yields per unit area. Bioethanol can be produced from various kinds of crops.

However, when ethanol yields per unit area of cultivations are compared, cassava is the highest potential crop to gain the highest yield. Average yields of cassava, sugar beet, sweet potato, wheat, sugar cane, rice, sorghum, and starchy corn are about 31.25, 56.00, 30.00, 9.00, 62.50, 7.31, 6.25, and 6.00 MT/ha/year (metric ton/ha/year), respectively. Carbohydrate contents (as starch or sugar) of those crops are approximately 28.0, 14.0, 24.5, 70.0, 10.5, 80.0, 70.0, and 70.0 (% w/w), respectively. Theoretical yields of bioethanol per unit area of cultivation per annum of those crops could be 4.95, 4.44, 4.16, 3.57, 3.53, 3.31, 2.48, and 2.38 MT/ha/year (tons of ethanol/ha/year), respectively. They were calculated from a ton of starch produces 566 kg of ethanol, and a ton of sucrose sugar produces 538 kg of ethanol.

Table I shows yields of different crops per unit area per year, starch or sugar content in each crop, theoretical yield coefficients, $Y'_{p/s}$ (mass of ethanol/mass of starch or sugar) produced stoichiometrically, and calculated yields of ethanol per unit area per annum.

Feedstocks (Crops)	Yield/rai (kg/rai)	Yield/ha (kg/ha)	No of crops (per year)	Yield/ha (MT/ha)	Yield/ha (MT/ha/year)	Starch or sugar content (%)	Starch or sugar (MT/ha/year)	Yield coefficient (Y'p/s)	EtOH/ha/year (MT/ha/year)	Grading
Cassava	5,000	31,250	1	31.25	31.25	28.0	8.75	0.566	4.95	a
Beet root	n/a	28,000	2	28.00	56.00	14.0	7.84	0.566	4.44	b
Sweet potato	n/a	15,000	2	15.00	30.00	24.5	7.35	0.566	4.16	bc
Wheat	n/a	4,500	2	4.50	9.00	70.0	6.30	0.566	3.57	c
Sugar cane	10,000	62,500	1	62.50	62.50	10.5	6.56	0.538	3.53	c
Rice	585	3,656	2	3.66	7.31	80.0	5.85	0.566	3.31	cd
Sorghum	500	3,125	2	3.13	6.25	70.0	4.38	0.566	2.48	e
Starchy corn	n/a	3,000	2	3.00	6.00	70.0	4.20	0.566	2.38	e

Notes:

– "Rai" is the Thai's unit area.; ha is hectare.; MT is metric ton.; EtOH means ethanol.; $Y'_{p/s}$ is theoretical yield coefficients of ethanol; where 0.566 and 0.358 g/g are from starch and sugar raw material, respectively.

– Yields per unit area of each crop in column 2 and 3 are the world's average yields from on-line searches (details not specified or shown herein).

– Percentages of starch or sugar content are in fresh feedstock for cassava, sugar beet, sweet potato, and sugar cane, and in dry feedstock for wheat, rice, sorghum, and starchy corn.

– Grading just differentiates the difference in their ethanol yields per unit area per year.
