# Supplementary material for: Single-step ethanol production from raw cassava starch using a combination of raw starch hydrolysis and fermentation, scale-up from 5-L laboratory and 200-L pilot plant to 3000-L industrial fermenters
Source: Biotechnol Biofuels. 2021 Mar 16;14:68. doi: 10.1186/s13068-021-01903-3 (PMC7962325; doi:10.1186/s13068-021-01903-3)
Supplement: Supplementary file 2 — Additional file 2. [file 13068_2021_1903_MOESM2_ESM.pdf]

Supplementary Material II

Degree of Starch Conversion, Hydrolysis of Starch to Release Glucose (molecules) and Its Yield Coefficient

The degree of conversion of raw starch to glucose was calculated as the percentage of glucose released from the raw cassava starch hydrolysis using the equation:

$$Degree\ of\ \ conversion\ (\%) = \frac{Glucose\ released\ (g/L)}{Raw\ cassava\ starch\ used\ (g/L) \times 1.11 (g/g)} \times 100 \qquad (1)$$

where 1.11 (g/g) is the 1.11 g theoretical (stoichiometric) yield of glucose from 1.00 g of starch, calculated from $\frac{n(C_6H_{12}O_6)}{(C_6H_{12}O_6)_n - ((n-1) \times H_2O)}$, where $C_6H_{12}O_6$ is the glucose with a molecular weight (MW) of 180.156 g/mol; H_2O is the water with a MW of 18.015 g/mol, and n is the number of glucose molecules of glucose and of starch or degree of polymerization (Dp). For example* (column 4, highlighted), if a starch chain polymer of 1,000 glucose molecules was completely hydrolyzed, where n = 1,000 molecules, then substitute into the above term to obtain $\frac{1{,}000(180.156)}{((180.156)_{1{,}000}) - ((1{,}000-1) \times 18.015)} = 1.11$ g/g. Although the n or Dp values vary (see low 2), the number of 1.11 g/g values is still obtained as a constant (see Table below).

Table I shows different starch polymers depending on their degree of polymerization (DP) or number of glucose molecules (n) and their molecular weights; glucose yields and their molecular weights; and theoretical yield coefficients, $Y'_{p/s}$ of glucose from each starch polymer hydrolysis.

Starch Polymers	Starch	Starch	Starch*	Starch	Starch	Starch
Degree of Polymerization, Dp or n/	Dp 10,000	Dp 5,000	Dp 1,000	Dp 500	Dp 100	Dp 50
Degree of Polymerization	10,000	5,000	1,000	500	100	50
MW (g/mol) of starch[S]	1,621,578	810,798	162,174	81,258	16,234	8,126
Hydrolysis to release glucose (molecules)	10,000	5,000	1,000	500	100	50
(MWof glucose of 180.156 x no. of glucose molecule)[P]	1,801,560	900,780	180,156	90,078	18,016	9,008
MW fraction = MW of glucose /MW of starch, (P/S)						
or the yield coefficient, $Y'_{p/s}$ (g/g)	1.11	1.11	1.11	1.11	1.11	1.11
One gram of starchs or their polymers relase						
glucose (per g)	1.11	1.11	1.11	1.11	1.11	1.11
On molar concentration basis						
Conclusions	One gram of starch produces 1.11 g of glucose.					

Note: Theoretical yield coefficient, $Y'_{p/s}$ is the stoichiometric yield of glucose produced per unit of starch hydrolyzed. The $Y'_{p/s}$ is calculated from P/S, where P is the (MWof glucose of 180.156 × number of glucose molecule) in row 6, and S is the MW of starch in row 4.
